# Fracture Load of an Orthodontic Appliance for Robin Sequence Treatment in a Digital Workflow

**DOI:** 10.3390/ma14020344

**Published:** 2021-01-12

**Authors:** Maite Aretxabaleta, Alexander B. Xepapadeas, Christian F. Poets, Bernd Koos, Sebastian Spintzyk

**Affiliations:** 1 Department of Orthodontics in the Centre of Dentistry, Oral Medicine and Maxillofacial Surgery within the University Hospital Tübingen, Osianderstr. 2-8, 72076 Tuebingen, Germany; alexander.xepapadeas@med.uni-tuebingen.de (A.B.X.); bernd.koos@med.uni-tuebingen.de (B.K.); 2 Department of Neonatology in the University Hospital Tübingen, Calwerstr. 7, 72076 Tuebingen, Germany; christian-f.poets@med.uni-tuebingen.de; 3 Section “Medical Materials Science and Technology”, University Hospital Tübingen, Osianderstr. 2-8, 72076 Tuebingen, Germany; sebastian.spintzyk@med.uni-tuebingen.de

**Keywords:** additive manufacturing, subtractive manufacturing, vat polymerization, SLA, DLP, rapid prototyping, time efficiency, digital dentistry, Tübingen palatal plate, orthodontic materials

## Abstract

CAD/CAM technologies and materials have the potential to improve the treatment of Robin Sequence with orthodontic appliances (Tübingen palatal plate, TPP). However, studies on the provided suitability and safety are lacking. The present study evaluates CAD/CAM technologies and materials for implementation into the workflow for producing these orthodontic appliances (TPPs), manufactured by different techniques and materials: additive manufacturing (AM) and subtractive manufacturing (SM) technologies vs. conventional manufacturing. The fracture load was obtained in a universal testing machine, and the breaking behavior of each bunch, as well as the necessity of adding a safety wire, was evaluated. The minimum fracture load was used to calculate the safety factor (SF) provided by each material. Secondary factors included manufacturing time, material cost and reproducibility. Dental LT clear showed the highest fracture load and best breaking behavior among AM materials. The highest fracture load and safety factor were obtained with Smile polyether ether ketone (PEEK). For the prototyping stage, the use of a Freeprint tray (SF = 114.145) is recommended. For final manufacturing, either the cost-effective approach, Dental LT clear (SF = 232.13%), or the safest but most expensive approach, Smile PEEK (SF = 491.48%), can be recommended.

## 1. Introduction

**Figure 1:**
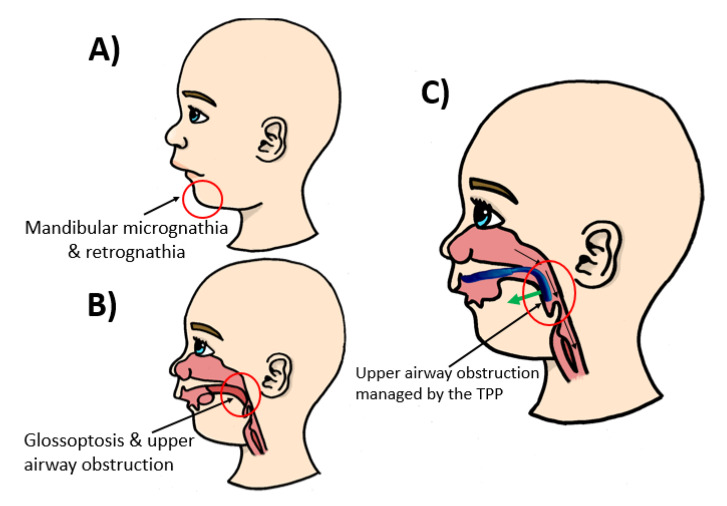
Anatomical condition and Tübingen palatal plate (TPP) positioning in patients with Robin sequence (RS). (**A**,**B**) Problems associated with the disease, such as mandibular retrognathia (receding jaw), micrognathia (small jaw) and glossoptosis (tongue placed further back than normal), which results in upper airway obstruction. (**C**) TPP solution for RS treatment and management of the airways by pulling the base of the tongue forward by a pre-epiglottic spur.

**Figure 2:**
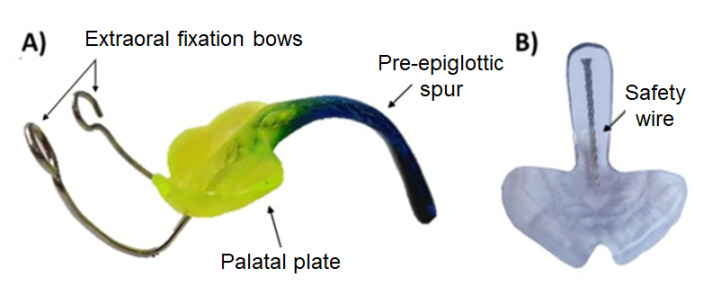
The Tübingen palatal plate (TPP). (**A**) An example of a finished conventional TPP with extraoral fixation bows. Custom color is added to the monomer–polymer mixture. (**B**) Printed transparent sample for showing the placement of the safety wire along the spur.

Robin sequence (RS) is considered a rare disease with an incidence of 1/8060 births in Germany [1,2]. Nonetheless, it needs to be treated right from birth. It is characterized by mandibular retrognathia, micrognathia and glossoptosis, as well as upper airway obstruction (Figure 1). Furthermore, RS is accompanied by a cleft palate in 80–90% of cases [3]. For this condition, the Tübingen palatal plate (TPP) is the treatment of choice at the University Hospital Tübingen [3,4,5,6,7,8]. This orthodontic appliance is shown in Figure 2. It consists of a palatal base plate covering the hard palate and cleft as well as the alveolar ridges, in order to support a velar extension or spur of individual length (approximately 3 cm; Figure 2), which is dorsally attached to the palatal plate and ends just above the epiglottis [3]. The TPP is able to relieve the upper airway obstruction by pushing the base of the tongue forward, thereby widening the pharynx [4]. The placement of the TPP is shown in Figure 1.

**Figure 3:**
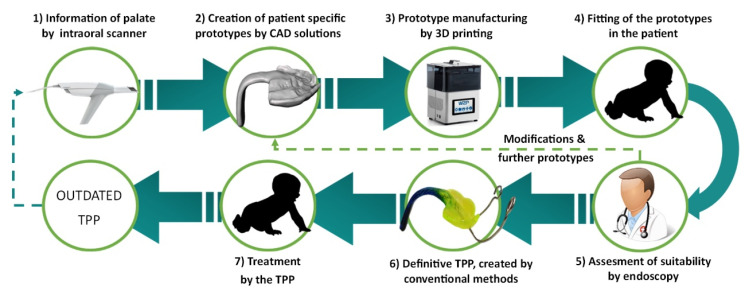
Current semi-digital workflow for the treatment of RS patients with the TPP.

The digital workflow for orthodontic treatment of RS and creation of the patient-specific prototypes were defined in a previous study and is the process currently in use at the University Hospital Tübingen [5]. A resume of the workflow is given in Figure 3. The process starts by obtaining information about the patient’s palate using an intraoral scanner. These data are then used to create different patient-specific prototypes by CAD software, where parameters such as spur angle and position are changed [5,9]. Afterward, these prototypes are printed with a direct light projection (DLP) printer (Solflex 170, Way2Production, Vienna, Austria) and a Medical Device Regulatory (MDR) class IIa splint material (V-Print splint, VOCO, Cuxhaven, Germany) [5]. The prototypes are placed in the oral cavity, and their suitability is checked in an endoscopy. This imposes a challenge as the material is transparent and does not offer a good contrast to the mucosa. The correct placement of the spur is just above the epiglottis on the frontal wall of the pharynx. The doctor needs to assess the suitability of these prototypes and, if necessary, modify them and validate the fit in another endoscopy. A standard length is used, which will be manually ground to adapt it to the patient.

To date, the final appliance is created by conventional manufacturing methods, duplicating the 3D printed prototype by duplication silicone and a cold polymerizing polymethylmethacrylate (PMMA) (Orthocryl clear, Dentaurum GmbH & Co. KG, Ispringen, Germany). Custom color is added to the monomer–polymer mixture to provide an optical contrast to the mucosa. In the definitive TPP, a threaded wire is added to the spur to increase stability, and extraoral fixation bows are polymerized into the palatal plate to counteract the force of the tongue base, as shown in Figure 2 [5]. As the patient grows, the TPP may become too small, so that the complete procedure must be repeated (Figure 3).

The threaded wire is incorporated to avoid parts chipping off in case of breakage and reduces the risk of these being swallowed by the patient (Figure 2). This is considered the gold standard. To date, however, the authors are not aware of any breakage of the appliance.

The above process is still time-consuming and prone to human error. The device occasionally shows some warping when transferring the prototype to the definitive TPP. Other studies have shown that acrylic resins are prone to shrinkage due to the polymerization process [10,11]. In addition, feedback from clinicians indicated that shrinkage might result in a worse fit of the TPP on the palate. In a previous study, it was pointed out that this issue could be avoided by having a complete digital workflow and manufacturing the final TPP by computer-aided design (CAD) and computer-aidedmanufacturing (CAM) technologies [5].

As an alternative to the current definitive TPP, materials with improved properties produced by CAD/CAM technologies may be used. Despite their potential to resolve some problematic aspects related to the current TPP workflow, insufficient information about the suitability and performance of such materials for use in a TPP exists. A study comparing different potential CAD/CAM materials to the conventional material has been carried out [12]. Five different additive manufacturing (AM) materials and two subtractive manufacturing (SM) materials were evaluated by a 3-point bending test. That study concluded that the tested CAD/CAM materials are not only comparable but exhibit superior mechanical properties compared to the conventional material. Although that study gives some insights into the mechanical properties, the performance in an actual TPP shape and related implications on the clinical application remains to be elucidated [12].

The present study aims to analyze the TPP’s mechanical performance in vitro and compare the break pattern of the materials in a more clinically relevant setup. The TPP must withstand the forces of the posterior tongue while pushing the base of the tongue forward without undergoing mechanical failure. The objective was to find a material that is safe enough to ensure no breakage in the patient, as well as a high manufacturing speed, an affordable price and a good optical contrast for the fitting procedure. Therefore, TPPs of the same materials as tested by Aretxabaleta et al. are evaluated in the present study in order to decide on the most appropriate material to be used in the future treatment of patients with RS [12]. Moreover, a colored CAD/CAM material for prototyping was considered. This could allow switching to a complete digital workflow. Moreover, the incorporation of a safety wire in the final TPPs produced by CAD/CAM was evaluated. Therefore, the following null hypotheses are considered in this study:

- There is no statistically significant difference in the fracture load of the tested CAD/CAM materials compared to the conventional solution,
- The addition of the wire does not increase the fracture load.

## 2. Materials and Methods

### 2.1. Sample Design and Selection

**Figure 4:**
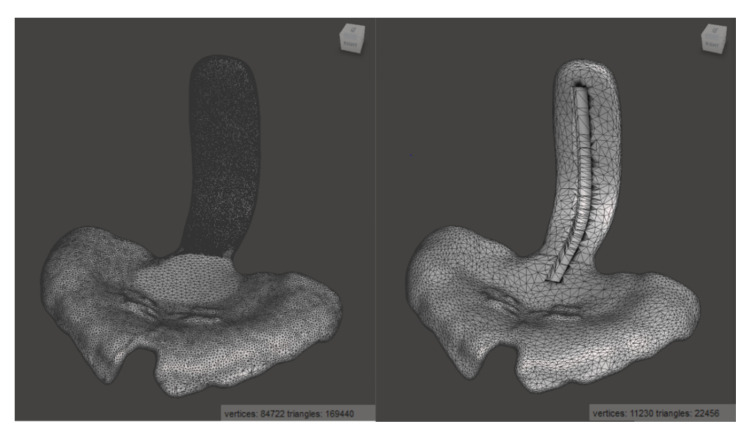
Standard TPP in Meshmixer, before (**left**) and after (**right**) mesh reduction and addition of the groove for the placement of the safety wire.

To create a realistic scenario, a TPP used in our clinical routine was taken as a standard sample for this study (Figure 4). For the creation of this TPP, the digital workflow based on the study of Xepapadeas et al. was used, which is the current procedure for TPP design and creation [5].

Samples were tested with and without stainless steel safety wire. The safety wire is a flat stainless steel threaded wire of 0.8 × 1.8 × 1 mm^3^ (Strengthener on coils, ref 312-106-00, lot 457619, Dentaurum GmbH and Co. KG, Ispringen, Germany) (Figure 2). The wire was added to the conventional TPP by removing material, placing the wire and polymerizing it with Orthocryl, whereas a different procedure was used for the CAD/CAM samples.

As the TPP STL file from the digital workflow contains too many triangles to be processed in Solidworks 2019 (Dassault Systemes SE, Vélizy-Villacoublay, France), the number was reduced from about 170,000 to 22,000 by using Meshmixer (Autodesk Inc., San Rafael, CA, USA) while avoiding to loose detail and with only a minimal reduction in accuracy (Figure 4). To preserve more detail in the area of the patient’s palate, a higher mesh reduction rate was used for the spur.

For incorporating the safety wire into the digital TPP, a groove of 1.2 × 2.2 × 4.2 mm^3^ was designed, which was trimmed from the shape of the spur by means of Solidworks, as shown in Figure 4. The groove was designed larger than the wire to ensure the remaining space for the addition of material when incorporating the wire into the specimen.

### 2.2. Manufacturing of Samples

As the TPP corresponds to a class IIa medical device according to the European Council Directive 93/42/ECC or European Medical Device Regulatory (MDR) 2017/745, only commercially available class IIa materials could be considered for the definitive TPP. This study employed the same technologies and materials as a previous study [12]. Both AM and SM technologies were taken into account, considering SLA (Stereolithography) and DLP for AM technologies. Moreover, identical devices, materials, software, as well as manufacturing and post-processing steps as in previous work were used [12]. For manufacturing and post-processing, the manufacturer’s instructions were followed.

Different DLP materials were taken into consideration: V-Print splint (lot #1846211, VOCO GmbH, Cuxhaven, Germany), Freeprint splint 2.0. (lot #211201, DETAX GmbH and Co. KG, Ettlingen, Germany) and Freeprint ortho (lot #220404, DETAX GmbH and Co. KG). Apart from these materials, one MDR class I material with blue color was considered for the prototyping phase: Freeprint tray (lot #04086, DETAX GmbH and Co. KG). Netfabb Premium 2019 (Autodesk, Mill Valley, CA, USA) was employed to prepare the printing files. Samples were manufactured by Solflex 350 (W2P Engineering GmbH, Vienna, Austria). For post-processing, specimens were placed in 98% Isopropanol (IPA) in an ultrasound cleaner (Ulsonix Proclean, Expondo GmbH, Berlin, Germany). All the materials were followed by a two-step ultrasound cleaning procedure. First, the samples were placed in an ultrasound bath with fresh IPA for 3 min, followed by a drying step using compressed air and finally, another 3 min ultrasound bath in fresh IPA (except V-Print splint, where the second cleaning is 2 min). Before post-curing, the samples were dried with compressed air and the support structures removed. Otoflash G171 (λ = 280–700 nm, NK-Optik GmbH, Baierbrunn, Germany) was used to post-cure all samples. Samples were cured twice with 2000 flashes. In between, they were turned over and left to cool-off for 2 min with the lid open. All samples were cured under nitrogen atmosphere, except for V-Print splint.

Dental LT clear V1 (lot #XK474N03, Formlabs, Sommerville, MA, USA) was the only SLA material that was considered in this study. Samples were manufactured by means of Form 2 (Formlabs). The printing file was prepared using PreForm V3.01. (Formlabs). Samples were oriented in the same position as the DLP samples. For post-processing, Formwash and Formcure devices (Formlabs) were used. The residual unreacted resin was cleaned by 98% IPA in a 20 min cleaning program in Formwash. Then, compressed air was employed to dry the samples. Finally, they were post-cured for 20 min at 80 °C using Formcure. Support structures were removed after complete polymerization.

In the case of SM, milled PMMA, Yamahachi PMMA D4 (lot #NC14 and #OL67, Yamahachi Dental MFG., Co., Gamagori, Japan) and Smile PEEK (lot #01133, Pressing Dental S.r.l., Falciano, Republica di San Marino) blanks with 25 mm thickness and 98.5 mm diameter were employed. Milling specimens were manufactured by the K5+ dry milling machine (vhf camfacture AG, Ammerbuch, Germany), where the milling job was prepared by vhf Dental CAM and Dental CNC software (vhf camfacture AG). “Denture” was selected as the milling strategy, and two milling tools were employed (vhf camfacture AG): P200-R2-40 and P100-R2-40.

**Figure 5:**
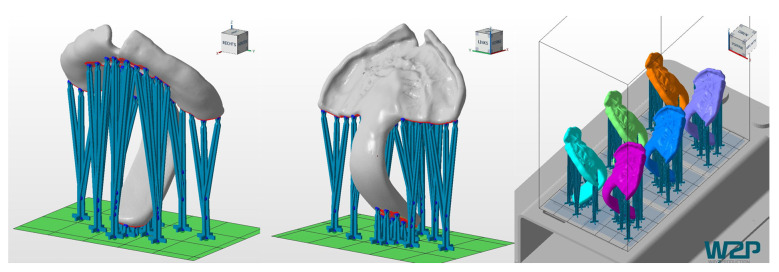
Printing job of the TPPs in Netfabb Premium 2019. Left and middle: TPP with support structures. Right: placement of the six TPPs in the building platform of the Solflex 350.

**Figure 6:**
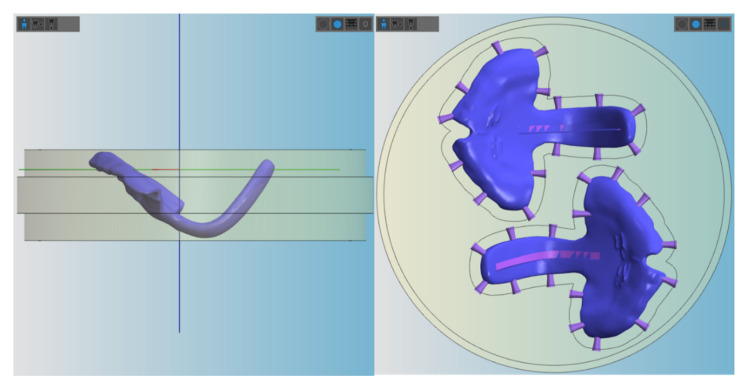
Placement of the TPP (with groove) in the blanks for milling in vhf Dental CAM. Left: positioning of the sample in the lateral view. Right: positioning of the samples from the top, where the connectors are shown.

All AM materials were printed in 100 µm, except for Freeprint tray, which could only be manufactured at a minimum layer height of 200 µm. AM samples (n = 6) were printed vertically, placing support structures, as shown in Figure 5. In contrast, SM samples were placed horizontally in order to fit in the defined blank thickness and connectors were situated as displayed in Figure 6.

Samples from the conventional TPP (gold standard) were manufactured using Orthocryl clear (Dentaurum) and a safety wire (stainless steel strengthener on coils, ref 312-106-00, lot 457619, Dentaurum). The material is a combination of liquid methylmethacrylate monomer (lot # 486569 A) and PMMA powder (lot # 480151 A).

A mold was created by duplicating 3D printed samples with a two-component duplication silicone (Dublisil ®, Dreve Dentamid, Unna, Germany) and filling the mold with Orthocryl clear. The recommended monomer to polymer ratio of 2.5:1 was too viscous to flow in the mold and was therefore reduced based on the technician’s experience. The samples were polymerized for 20 min at 40 °C and 2.2 bar in a pressure pot (Polymax 5, Dreve Dentamid, Unna, Germany) and then removed from the mold. A groove was manually created in the spur, and a 4 mm long safety wire was added and polymerized as described above. The remaining inaccuracies are sanded down to obtain the expected spur thickness.

For the CAD/CAM materials, two different processes were used for the addition of the wire, one for the AM and one for the SM materials. For the AM materials, the wire was incorporated between post-rinsing with IPA and post-curing. Each wire was trimmed into 4 cm segments and pre-bent to shape manually. One wire was placed into each groove, gradually filling the space with the corresponding resin using a syringe with a microtip. The deposited thin layers of material were cured for 10 s each with a LED-UV curing device (Bluephase Style 20i, Ivoclar Vivadent AG, Schaan, Liechtenstein). The process was repeated until the groove was filled and the wire secured inside. The finished samples were then placed in the corresponding UV-curing device and cured following the manufacturer’s instructions.

TPP samples with and without wire manufactured by AM technologies with major fabrication errors, such as bubbles or cracks, were rejected. In the case of the SM materials, the wire was only added to Yamahachi PMMA as there were no concerns for the stability of PEEK, and it had shown a highly ductile behavior as well as no breakage in a previous study [12]. The STL file with the groove was milled, and the wire was prepared as described before and polymerized into the spur by adding Orthocryl (20 min; 40 °C; 2.2 bar in a pressure pot). Overflown and cured material was sanded down manually until the spur had the expected thickness.

**Figure 7:**
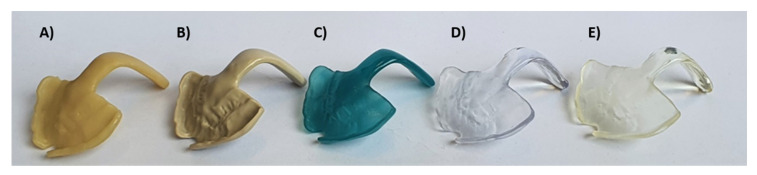
Examples of some of the tested TPPs of different computer-aided design (CAD)/CAM materials (without wire). SM materials: (**A**) Yamahachi PMMA color D4, (**B**) Smile PEEK; AM DLP materials: (**C**) Freeprint tray, (**D**) V-Print splint; AM SLA material: (**E**) Dental LT clear.

Hereby, the gold standard could be compared to TPPs produced by all studied CAD/CAM materials with and without wire. The different samples used for the testing are shown in Figure 7 (Freeprint splint and Freeprint ortho have a similar color to V-Print splint and are therefore not shown in the image).

### 2.3. Time Efficiency and Affordability of Materials

The time needed for creating and manufacturing the standard TPP used in this study was measured for the following steps: scanning the patient, designing the patient-specific TPP, preparing the workfile, preparing the printer or milling machine, manufacturing process, post-processing of the material and manual/final post-processing (if applicable). While the timing was fairly constant in the CAD/CAM process, it was more difficult to divide it for the conventional process. Therefore, only the steps of information acquisition from the patient (by a conventional maxillary impression) and manufacturing of the plate were taken into account.

The other relevant aspect is the material cost. In the case of Orthocryl and AM materials, values were calculated based on the volume of the standard TPP and the cost of each material. In the case of AM material, the volume of the used supports was also considered. For Orthocryl, the recommended monomer to polymer ratio was used. Finally, the price of the SM materials was calculated by dividing the price of each blank by the number of TPPs, which can be manufactured from it (two standard TPPs).

### 2.4. Measurement of Fracture Load of Standard TPPs

With the aim to simulate the clinical environment, samples were kept in distilled water (Quality 1, ISO 3696) at 37 ± 1 °C for 50 ± 1 h, following the procedures of Aretxabaleta et al. [12]. Before testing, the specimens were placed in a water container with distilled water at room temperature 23 ± 2 °C. Specimens were dried using a paper towel and directly measured in dry conditions.

**Figure 8:**
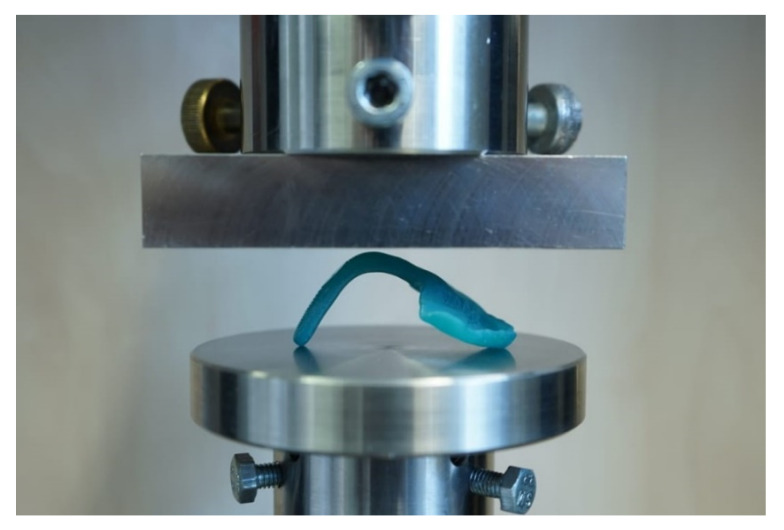
Test setup for TPP test with inserted test specimen in the universal testing machine.

For testing, the TPP samples with and without wire were positioned flat with two contact points at the front part of the palate and one contact point at the apical end of the spur. Hereby, the force of the load plate was applied in the curvature of the orthodontic appliance (Figure 8). All tests were performed in a universal testing machine (Zwick Z010, Zwick GmbH, Ulm, Germany) with the following measuring parameters [12]: 10,000 N load cell, crosshead speed of 2 mm/min and a preload force of 1 N. The maximum deflection of 14 mm was set in the machine.

With this setup (Figure 8), the maximum force at breakage and the maximum deflection at breakage were measured. Moreover, the behavior of the material to the applied force or the breaking pattern could be evaluated.

Information such as the break pattern is an important safety factor, which needs to be taken into account for the clinical application. The factors to consider for avoiding the risk of material swallowing are the material breaking off into smaller pieces or spur completely breaking off. Therefore, in this study, the materials’ behavior was classified into four different patterns or types, from the best (A) to the worst scenario (D): No breakage of the sample (type A), breakage, but no detachment from the wire (type B), breakage and partial detachment from the wire (type C), and total breakage and/or detachment of the wire from the base of the palatal plate (type D). For obtaining a suitable and safe TPP, types A and B were deemed acceptably safe for samples with wire. In contrast, only type A was considered acceptable for samples without wire.

In addition, the safety margin provided by each group was calculated. For that, the lowest measured force value from each group was compared against a minimum force requirement. The authors are not aware of any studies dealing with the force that an RS infant’s tongue may exert on the spur of the TPP, even though a study dealing with forces exerted in the posterior side of the tongue of healthy adults was found. Sommer et al. measured the maximum force of the human tongue in a posterior isometric direction, obtaining values in the range of 3.2–52.1 N (14.1 ± 7.5 N) [13]. The values obtained from this study can help to put the force values of the tested TPPs into perspective, which is important for the design and performance of the appliance. Using the maximum value of 52.1 N recorded in adults, the minimum force required for the TPP was set.

### 2.5. Statistical Analysis

Statistical analyses were performed using JMP 14 (SAS, Cary, NC, USA). The normal distribution of the data was checked using the Shapiro–Wilk test. In the case of the normal distribution, a One-way ANOVA was employed to compare means and evaluate the effect of the wire within all groups, except for PEEK. As a post hoc parametric analysis using a paired Student’s t-test was performed for all groups (including all materials with and without wire reinforcement). A *p*-value of <0.05 was considered statistically significant.

## 3. Results

### 3.1. Time Efficiency and Affordability of Materials

An approximation of the time needed by each process to manufacture the standard TPP without wire is shown in Table 1. The fastest technique is the manufacturing by SM, followed by the traditional and finally the DLP technologies. For the addition of the wire, extra time was necessary: 7 min for AM and 30 min for SM materials. The increased time for SM materials was due to the necessary steps to add Orthocryl into the slot, as well as keeping the samples in a pressure pot for 20 min and another 10 min for the finishing steps. The same occurs when placing the wire in the conventional samples, adding an additional 35 min to the process.

The price of each TPP may vary depending on the used manufacturing technique and material. The cost per TPP of the studied materials is shown in Table 2, in which the material cost of the studied standard TPP is given (Figure 4). When comparing the material costs, the SM materials were the most expensive ones, especially the Smile PEEK. Opposed to that, the conventional material was the cheapest, followed by DLP materials (Freeprint tray, Freeprint splint, Freeprint ortho, V-Print splint).

### 3.2. Measurement of Fracture Load of Standard TPPs

**Figure 9:**
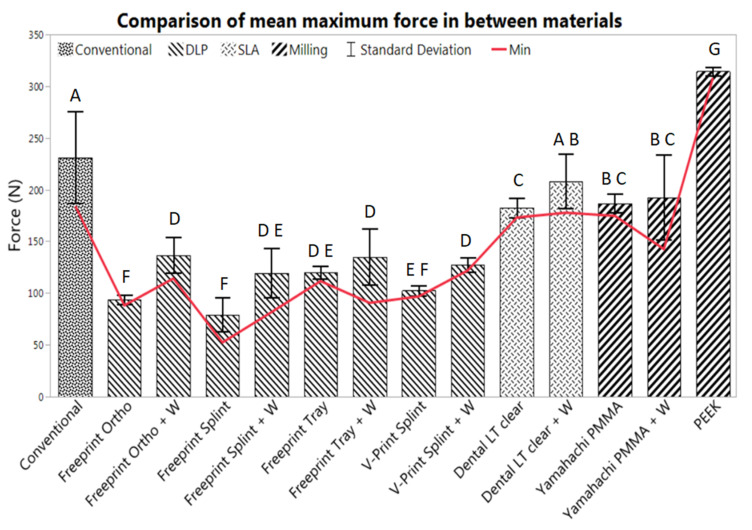
Mean max force (n = 6) and standard deviation, obtained from the TPPs manufactured by all evaluated materials with (+ W) and without wire. The conventional solution (Orthocryl + W) was compared to AM materials (middle group) and SM materials (right group). The letters denote the results of the paired Student’s t-test with a *p*-value of <0.05.

A total of six TPP samples were tested per material. Values of maximum exerted force and maximum deflection were obtained for all samples. In Table 3, the average values for the maximum exerted forces and maximum deflection are shown, as well as the number of specimens undergoing mechanical failure and the samples that did not break when testing. In Figure 9, all possible combinations are compared to the conventional solution.

The conventional material (Orthocryl with wire) had an average maximum force value of 231.0 ± 44.5 N (*p* = 1.0) and a maximum deflection of 6.9 ± 4.5 mm (Figure 9). All samples involving the gold standard underwent mechanical failure.

All tested AM and SM groups exhibited normal distribution. Most showed a statistically significant difference to the conventional solution (*p* < 0.05), except for Dental LT clear with wire (*p* = 0.0715).

When it comes to AM materials, most showed a significantly lower fracture load than the conventional one. All AM materials suffered from breakage, except for some samples using Dental LT clear with and without wire. The SLA material showed superior flexural strength among AM materials. Dental LT clear without wire had a maximum force of 174.5 ± 1.4 N and a maximum deflection of 12.5 ± 1.2 mm for the break scenarios and a maximum force of 190.4 ± 4.2 N and a maximum deflection of 14 mm for the no-break scenarios (Table 3). These values were higher than all the other DLP materials with and without wire. Moreover, the results show a statistically significant increase in the maximum applied force when using the reinforcement wire for almost all AM materials (*p* < 0.05), except for Freeprint tray (*p* = 0.2368) and while having the largest impact on Freeprint ortho (with an increase of 45.27%) and Freeprint splint (with an increase of 51.11 %) (Table 3). Dental LT with wire showed a comparable value to the conventional solution (*p* = 0.0715), obtaining a maximum force value of 200.6 ± 20.7 N for the break scenario and 246.1 N for the only sample not suffering breakage. Despite the fact that average values from the Dental LT clear without wire were significantly lower (*p* < 0.05) than those obtained with the gold standard as well as Dental LT with wire, much higher average deflection values and smaller standard deviations were obtained (Table 3, Table 2).

Regarding the SM materials, all samples from Yamahachi PMMA broke, whereas none of the Smile PEEK samples underwent mechanical failure. Yamahachi PMMA showed an average maximum force of 186.6 ± 9.1 N, increasing to 192.7 ± 41.3 N with wire. Despite this, the increase of force by the addition of the wire was not statistically significant (*p* = 0.6290). Instead, the maximum deflection was reduced when the wire was added to the Yamahachi samples, from 5.8 ± 0.5 mm to 4.3 ± 1.0 mm. Both values were significantly lower (*p* < 0.05) than the values obtained by the conventional solution (Table 3). PEEK samples showed significantly higher force (314.3 ± 3.9 N) and deflection values compared to the conventional one (*p* < 0.05), as well as the rest of the evaluated materials. PEEK samples exhibited a ductile behavior and reached the limit of 14 mm preset in the machine. Moreover, both SM materials showed smaller standard deviations than the gold standard for the force and deflection values (Table 3). In the case of Yamahachi PMMA, the standard deviation was higher in the samples with wire in comparison to those without.

**Figure 10:**
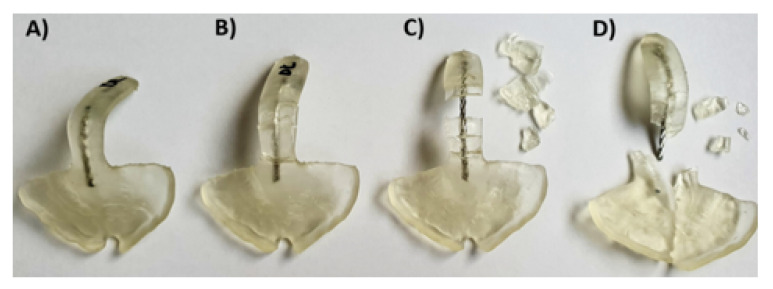
Different breaking patterns were observed in TPPs with wire from the ideal case (left) to the worst scenario (right) in Dental LT clear SLA material. (**A**) Ideal case, no cracks after application of the force; (**B**) sample broke, all pieces attached to the wire; (**C**) sample broke, the biggest parts still attached to the wire, smaller ones detached from the wire; (**D**) worst scenario, where the sample broke and the safety wire, as well as all pieces, are completely detached from the palatal plate and go further in the pharynx of the patient.

**Figure 11:**
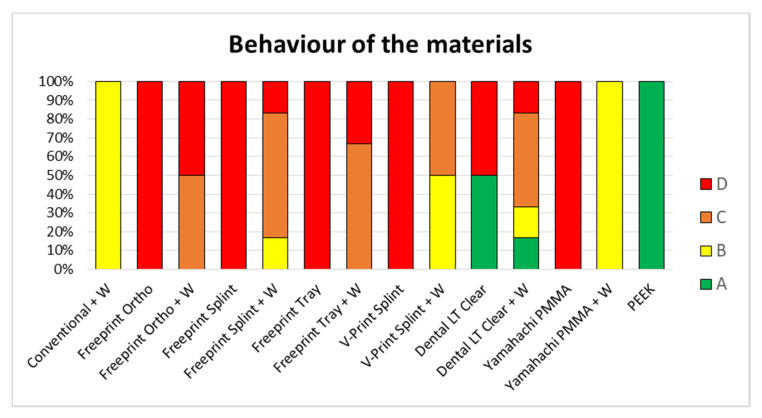
Classification of the behavior of the different samples with (+W) and without wire to the testing (n = 6). Classification from the ideal (A) to the worst scenario (D). The conventional solution (Orthocryl + W) is compared to AM materials and SM materials. Different AM materials can be found: Dental LT clear (SLA), Freeprint tray (DLP), Freeprint ortho (DLP), Freeprint splint (DLP) and V-Print splint (DLP). Furthermore, different SM can be found; Yamahachi PMMA and Smile PEEK.

Figure 10 shows a resume of the different behavior of the samples in the test setup. Type B and C could only be found in samples without wire. Therefore, only type A was considered acceptable for the samples without wire. Following this criterion, samples were classified into different types (Figure 11). This classification needs to be taken into consideration, together with the values shown in Table 3.

The gold standard exhibited type B in all specimens (Figure 11). In almost all DLP specimens, a type C or D break was observed (Figure 11). Exceptions were Freeprint splint and V-Print splint materials, as the addition of the wire improved their behavior to a type B, which had a higher impact on the latter (Figure 11). Regarding the SLA material (Dental LT clear), diverse break patterns and reactions to the applied force were observed. In the specimens without wire, two different behaviors were observed, type A and D. Instead, when the wire was added, all possible types were observed (Figure 10), C being the most probable. Despite the observed forces for the Dental LT clear being statistically higher when the wire was added (Table 3, Figure 9), the break pattern behavior was worsened, as the ideal type, A, was obtained less often.

Concerning the SM materials, type A was observed for all PEEK samples, as it did not undergo any mechanical failure (Figure 11). Moreover, high average maximum force and deflection values, as well as a small standard deviation, were observed for all samples (Table 3, Figure 9). For Yamahachi PMMA, two different behaviors were observed, type D for all samples without wire and type B for all the samples with wire (Figure 11).

The minimum force that the TPP must withstand was set at 52.1 N in order to guarantee sufficient safety. Table 4 shows the safety margin (%) that each tested sample offered in relation to the minimum requirement (52.1 N). The highest safety margin was provided by Smile PEEK, as it provides a safety margin of almost 500%. This was followed by the conventional solution with an increase of 253.54%, Dental LT clear + W with 241.09%, Yamahachi PMMA with 235.27% and Dental LT clear with 232.13%. The rest of the combinations offered an increase of less than 200% in respect to the lowest recorded force value, where DLP materials with and without wire provided a lower percentage of increase compared to the milled PMMA.

## 4. Discussion

Each manufacturing technology has its own limitations and advantages where materials, their quality, built volume, manufacturing speed and post-processing protocol play a major role [14]. For an efficient application in a clinical setting, time, and expense are crucial factors as well. The provided durations included the entire process for manufacturing TPPs (Table 1), where the workload for the technician differs. While the value refers to the actual work time in the conventional process, the hands-on time in the CAD/CAM processes is shorter.

Despite DLP appearing to be the slowest technique, this information is not accurately representing the potential of this technique (Table 1). The recorded timing was obtained for the Solflex 350. From experience, it is known that other DLP printers such as Solflex 170 (W2P Engineering GmbH, Vienna, Austria) and Rapidshape D30II (Rapid Shape GmbH, Heimsheim, Germany) allow faster printing speeds. Therefore, the values shown in Table 1 may differ when other devices are used. In contrast to the SLA and SM processes, the time is not dependent on the number of parts in the building platform for DLP (Figure 5), as it can create them simultaneously. When it comes to the SLA and SM processes, the manufacturing time is dependent on the number of produced parts, which will prolong the prototyping phase.

For conventional manufacturing, the whole process is strongly dependent on the level of expertise of the dental technician. Furthermore, it is important to underline that for a safe application, the manufacturer requires the immersion of the Orthocryl parts in water for three days to reduce the residual monomer content [12]. For CAD/CAM technologies, the scanning and designing phase, as well as the post-processing, can be influenced by the user. Additionally, if intraoral scanning is implemented in the conventional manufacturing workflow, the information acquisition time is similar to the CAD/CAM processes. Once the virtual model of the palate is created, it needs to be manufactured by CAD/CAM, a technology (DLP or SLA) requiring additional time, which should be considered in the values shown in Table 1.

The material cost was highest for SM (Table 2). One reason is the higher material waste as opposed to AM, where only the volume of the part is employed. Apart from being cheaper, AM technologies offer a more environmentally friendly solution as they do not waste as much material.

It is important to underline that for this study, a clinically relevant build orientation was employed in all technologies. Samples were positioned so that no support structures were placed in the palatal area, where a higher degree of accuracy is needed. Instead, it was considered preferable to position them in areas that will be post-processed later on (Figure 5 and Figure 6). This build orientation is known to significantly influence the process and fabrication attributes, such as manufacturing time, part cost and surface finish [15]. Therefore, the obtained values are largely dependent on the used build orientation.

Concerning timing and efficiency, DLP is the most suitable technology for the prototyping phase, as multiple TPPs can be manufactured at once, fast and inexpensively. For the definitive TPP (only one appliance being produced), the results of the mechanical testing play a decisive role, while the above-mentioned factors are only secondary.

Despite no standard measurement method existing in the literature, the first information on the performance of different CAD/CAM materials for TPP production in an in vitro setup could be evaluated. Other studies also show that using a simplified in vitro approach can give valuable information on the clinical situation [16]. Besides the test setup, printing orientation is known to influence the results of the mechanical testing. Considering the printing orientation and the positioning in the used test setup, the printed layers are oriented in parallel to the applied force. In this scenario, the facture load or force can be expected to be lower than when they are perpendicular [15,17]. Moreover, this study aimed to mimic in vivo conditions by preconditioning the samples in distilled water at 37 ± 1 °C for 50 ± 2 h. Storage medium, as well as temperature, are known to have an influence on the properties of materials. The plasticizing effect of water, which is dependent on their degree of water absorption, might decrease the fracture load value [12]. Moreover, placement of supports was avoided in the spur area as much as possible (Figure 5 and Figure 6), considering that it can create weak spots potentially influencing the break pattern and maximum force.

The authors are not aware of any studies dealing with the force that a RS infants’ tongue may exert on the spur of the TPP. Therefore, the maximum force of the human tongue in a posterior isometric direction was taken as a standard. Sommer et al. obtained values in the range of 3.2–52.1 N (14.1 ± 7.5 N). The authors stated that “although the maximum measurement of 52.1 N may represent an extreme case, this kind of force should be considered for the future conception and development of tongue management systems” [13]. In particular, the study pointed out that this value needs to be taken into account for the design and performance of tongue restraining devices used in patients suffering from obstructive sleep apnea (OSA), which is also part of the clinical picture in RS patients. In OSA patients, retraction of the posterior side of the tongue results in an upper airway collapse. Some limitations of the study include that the tongue was slipping from the measuring setup and that the technique was painful for participants, which may have led to an underestimation of the maximum force (52.1 N in an adult male) actually possible [13]. Taking into account that this value was obtained in adults, it is expected to be considerably lower for the present application, even though the concern of obtaining enough safety is primordial in newborns. In contrast to adult OSA patients, newborns do not show involuntarily coughing out fragments when a break or fracture occurs.

Only Dental LT with wire showed a comparable fracture load to the conventional solution (*p* = 0.0715). Most of the materials showed significantly lower values, apart from the SMmaterials. Therefore, the first null hypothesis was rejected for most materials, as the fracture load is not comparable to the conventional one. The vast majority of the TPPs without wire underwent mechanical failure (case D), where the spur was completely separated from the base plate (Figure 11). In the case of the Dental LT clear, only half the samples underwent mechanical failure. Even though, significantly lower fracture load than with the conventional solution was obtained. Opposed to that, Smile PEEK did not break in any of the samples (case A) and exhibited a significantly higher fracture load. Considering the previous study, Dental LT clear and Smile PEEK exhibited a more ductile behavior compared to the other tested materials, Smile PEEK being the most ductile [12]. This could explain the high forces potentially endured without breaking, as well as the high deflection values (Table 2).

Despite an improved breaking behavior for both AM and SM materials with the addition of the wire was seen, this increase only yields to an improvement up to case B in the best scenarios (Yamahachi PMMA and V-Print splint) (Figure 11). Apart from that, the obtained mean fracture load value is significantly increased for most of the samples, except for Freeprint tray and Yamahachi PMMA (Table 3, Figure 9). Therefore, the second null hypothesis that the fracture load is not increased must be rejected for most of the AM materials while being accepted for Freeprint tray and Yamahachi PMMA.

The AM TPPs contain more manufacturing steps where the mechanical properties of the material can be influenced by the user [12], e.g., during post-processing or by some manufacturing factor, such as the condition of the material, printing failures, bubbles, degradation of the printing vats, cleaning level of the IPA after some uses, proper functioning of the UV curing machines, etc. Nonetheless, the manual attachment of the safety wire and manual curing of AM samples can result in a non-reproducible and more complicated process that usually leads to weak spots in the spur (such as bubbles), as well as non-uniform curing and mechanical properties. Therefore, adding the wire can be recommended for materials with a low safety margin (Table 4) in combination with a significant increase of fracture load (Table 3, Figure 9), specifically V-Print splint, Freeprint splint and Freeprint ortho. Meanwhile, a wire should be avoided in the Freeprint tray and Dental LT clear.

While the manufacturing process of conventional and AM parts consists of more manual steps which are more prone to human error, the mechanical properties of SM parts are less influenced by manufacturing. SM materials offer process stability and reproducibility [12]. By manual addition of the wire, these qualities can be compromised, and the manufacturing time is increased (Table 3). Despite the improved breaking pattern of the material (case B) with the addition of the wire (Figure 11), there is no significant increase in force (Figure 9) and even the safety margin is measurably decreased by 61.74% (Table 4). Moreover, the process for wire addition is not reproducible, and the bond between the Orthocryl filling and Yamahachi PMMA may not be uniform, which can explain the higher standard deviation for those samples (Table 3, Figure 9). Therefore, the wire addition cannot be recommended for Yamahachi PMMA.

Taking into account the safety margin (Table 4), the mechanical properties of the materials obtained in the previous study [12], the break pattern in the clinical setup (Figure 11), as well as the information of time (Table 1) and cost (Table 2), two recommendations on the technology and material to use in the future can be made. DLP is the most suitable technology to be employed for the prototyping stage, as it can produce more prototypes in less time than the other technologies. The high contrast material Freeprint tray can withstand higher forces in comparison to the other DLP splint materials (Table 3). Even without wire, the value is high enough to be recommended (Table 4). It has a comparable fracture load compared to the V-Print splint (Figure 9), which is the material currently used for TPP prototypes in the clinics [5]. As time is a crucial factor at this early stage of treatment, and the fitting occurs under endoscopic supervision in a controlled environment, the use of a Freeprint tray without wire can be recommended. Whether the printing layer height of 200 µm has an influence on the success of fitting the TPP remains to be evaluated. When it comes to the definitive appliance, SLA and SM materials are recommended. Smile PEEK is the safest material, but also the most expensive one. On the opposite, Yamahachi PMMA and Dental LT clear offer a high safety margin even without safety wire, along with a more affordable price. While the flexural strength of both materials was found to be similar, Dental LT clear shows a more ductile behavior [12]. Furthermore, it showed less breakage in the clinical setup (Figure 11) and proposes a cheaper solution. Moreover, the post-processing for SLA is more reproducible and less prone to human errors, as it is more automatized than the one for DLP. Therefore, after the SM materials, Dental LT clear offers the most reproducible and stable mechanical properties. Smile PEEK and Yamahachi also meet the safety requirements, although with additional material cost.

To mention some limitations of this study, the provided timing is user, tool and technology-dependent, and does only reflect a selection of materials and CAD/CAM technologies available on the market. When it comes to the costs of the parts, only the material’s price was taken into account, not considering the cost of labor, machines, maintenance, tools, electricity, etc., in the final price. Apart from that, the fracture load of the TPPs was measured in a simplified approach, and therefore, the obtained values can be seen as a close approximation. While not accurately replicating the real force that will be exerted on the part inside the patient, it gives a first estimation of the amount of force required to break this orthodontic appliance. The values are expected to differ depending on spur angulation and thickness. However, the setup allows deciding for a material in a direct comparison. Nevertheless, only a selection of potential and commercially available materials and manufacturers was considered in this study.

## 5. Conclusions

In this study, potential CAD/CAM (AM and SM) materials were compared to conventionally used material (Orthocryl), and their suitability for manufacturing TPPs in a complete digital workflow was analyzed. Considering the included factors of safety, manufacturing workflow, material price, manufacturing time, reproducibility and mechanical behavior under load, the current study recommends solutions for the prototyping stage and the manufacturing of the definitive appliance. For prototyping, DLP was found to be the most appropriate technology to be employed, and the Freeprint tray offered one of the highest safety margins, alongside color contrast. For manufacturing the definitive TPP in a digital workflow, the use of Dental LT clear is encouraged, as it offers one of the highest safety margins as well as the most affordable solution. Yamahachi PMMA and Smile PEEK also met the safety requirements and seemed safe enough for the application, although manufacturing can be significantly more costly in the case of PEEK. Therefore, the choice is to use a safe, cost-effective approach, such as Dental LT clear or going for the most expensive but also the safest solution, Smile PEEK.

## Figures and Tables

**Table 1:** Approximation of the timing for creation and manufacturing of a single TPP (without wire) by the different techniques. Time for the different steps of the CAD/CAM technologies was divided also measured: scanning of the patient (S), designing of the TPP (D), preparing the manufacturing workfile (PW), preparing the printer/milling machine (PP), manufacturing (M), post-processing printer (PP-P), manual post-processing (PP-M).

Technology	Information		Manufacturing of the TPP					Total Time
**Conventional**	20 min		2 h 30 min					2:50:00
**CAD/CAM**	**S**	**D**	**PW**	**PP**	**M**	**PP-P**	**PP-M**	
SLA	2 min	45 min	40 s	2 min	1 h 52 min	1 h 20 min	15 min	4:16:40
DLP			4 min 20 s	5 min	2 h 50 min	17 min	15 min	4:21:20
Milling			1 min 30 s	1 min 30 s	1 h 30 min	/	2 min	2:22:00

**Table 2:** Material costs per TPP (without wire) lowest to highest.

Material	Material Cost Per TPP (€)
Conventional	0.44 €
Freeprint tray	0.93 €
Freeprint ortho	0.95 €
Freeprint splint	0.95 €
V-Print splint	1.40 €
Dental LT clear	2.57 €
Yamahachi PMMA	21.47 €
Smile PEEK	110.00 €

**Table 3:** Mean maximum force (N) and mean deflection (mm) values. Results of specimens with (+W) and without wire are shown, as well as the number of samples for each scenario (break or no break).

Samples ^1^		Number of Samples	Mean Maximum Force (N) ± Std.	Increase by Wire (%)	Mean Deflection (mm) ± Std.
Break	Conventional + W	6	231.5 ± 44.5	/	6.9 ± 4.5
	Dental LT clear	3	174.5 ± 1.4	15.00%	12.5 ± 1.2
	Dental LT clear + W	5	200.6 ± 20.7		10.5 ± 1.6
	Freeprint ortho	6	94.0 ± 4.4	45.27%	6.8 ± 1.3
	Freeprint ortho + W	6	136.5 ± 17.2		9.2 ± 3.2
	Freeprint splint	6	79.1 ± 16.4	51.11%	9 ± 0.6
	Freeprint splint +W	6	119.5 ± 23.6		6.0 ± 2.7
	Freeprint tray	6	120.0 ± 6.3	12.40%	7.3 ± 1.0
	Freeprint tray + W	6	134.9 ± 27.2		6.3± 2.5
	V-Print splint	6	102.3 ± 4.6	24.36%	9.6 ± 1.3
	V-Print splint + W	6	127.2 ± 6.7		8.8 ± 1.6
	Yamahachi PMMA	6	186.6 ± 9.1	3.24%	5.8 ± 0.5
	Yamahachi PMMA + W	6	192.7 ± 41.3		4.3 ± 1.0
No Break	Dental LT clear	3	190.4 ± 4.2	29.21%	14.0
	Dental LT clear + W	1	246.1		14.0
	PEEK	6	314.3 ± 3.9	/	14.0

^1^ Specimens with (+ W) and without wire, as well as number of samples for each scenario (break or no break).

**Table 4:** Provided safety margins (%) for samples with (+W) and without wire from highest to lowest.

Sample	Safety Margin (%) ^1^
Smile PEEK	491.48
Conventional + W	253.54
Dental LT clear + W	241.09
Yamahachi PMMA	235.27
Dental LT clear	232.13
Yamahachi PMMA + W	173.53
V-Print splint + W	133.85
Freeprint ortho + W	119.09
Freeprint tray	114.14
V-Print splint	85.63
Freeprint tray + W	73.79
Freeprint ortho	69.16
Freeprint splint + W	56.53
Freeprint splint	0.29

^1^ Difference between the lowest measured fracture force value of each group to the minimum safety requirement (52.1 N).

## Data Availability

The data that support the findings of this study are available from the corresponding author, Maite Aretxabaleta, upon reasonable request.
